# A sensitive LC–MS/MS method for the quantification of the plant toxins hypoglycin A and methylenecyclopropylglycine and their metabolites in cow’s milk and urine and application to farm milk samples from Germany

**DOI:** 10.1007/s00216-023-04607-9

**Published:** 2023-03-06

**Authors:** Ahmed H. El-Khatib, Julika Lamp, Stefan Weigel

**Affiliations:** 1 Department for Safety in the Food Chain, German Federal Institute for Risk Assessment (BfR), Max‑Dohrn‑Str. 8‑10, 10589 Berlin, Germany; 2 Department Safety and Quality of Milk and Fish Products, Max Rubner-Institut, Federal Research Institute of Nutrition and Food, Hermann-Weigmann-Str. 1, 24103 Kiel, Germany

**Keywords:** Sycamore maple (*Acer pseudoplatanus*), Methylenecyclopropylacetyl-glycine, Methylenecyclopropylformyl-glycine, Methylenecyclopropylacetyl-carnitine

## Abstract

Hypoglycin A (HGA) and its homologue methylenecyclopropylglycine (MCPrG) are present in ackee and lychee as well as seeds, leaves, and seedlings of some maple (*Acer*) species. They are toxic to some animal species and humans. The determination of HGA, MCPrG, and their glycine and carnitine metabolites in blood and urine is a useful tool for screening for potential exposure to these toxins. In addition, HGA, MCPrG, and/or their metabolites have been detected in milk. In this work, simple and sensitive ultra-performance liquid chromatography tandem mass spectrometry (UPLC–MS/MS) methods without derivatization were developed and validated for the quantification of HGA, MCPrG, and their metabolites in cow’s milk and urine. An extraction procedure from milk samples has been developed, whereas a dilute-and-shoot approach was implemented for urine samples. For quantification, the MS/MS analysis was performed in multiple reaction monitoring mode. The methods were validated according to the European Union guidelines using blank raw milk and urine as matrices. The limit of quantification presented here for HGA in milk (1.12 µg/L) is noticeably lower than the lowest published limit of detection (9 µg/L). Acceptable values for recovery (89–106% and 85–104% in milk and urine, respectively) and precision (≤ 20%) were obtained for all the quality control levels. The stability of HGA and MCPrG in frozen milk over a period of 40 weeks has been demonstrated. The method was applied to 68 milk samples from 35 commercial dairy farms and showed the absence of any quantifiable amounts of HGA, MCPrG, and their metabolites.

**Graphical Abstract:**

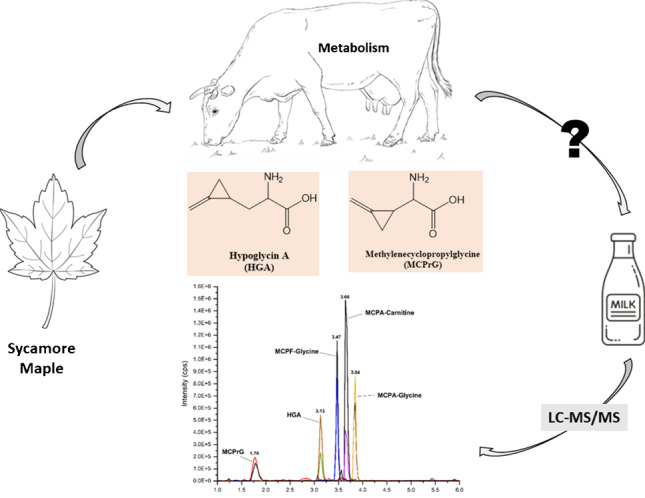

**Supplementary Information:**

The online version contains supplementary material available at 10.1007/s00216-023-04607-9.

## Introduction

Hypoglycin A (HGA, methylenecyclopropylalanine) and its homologue methylenecyclopropylglycine (MCPrG) are naturally occurring non‐proteinogenic toxic amino acids of plant origin [1]. These amino acids are abundantly formed in some plants of the family Sapindaceae such as lychee and ackee (especially in unripe fruits) [2–6] as well as in the seeds, leaves and seedlings/young shoots of some maple trees (*Acer* species) including sycamore maple (*Acer pseudoplatanus*) [7–11] and box elder maple (*Acer negundo*) [11, 12]. HGA and MCPrG are known to be toxic to many species, and their consumption has been associated with outbreaks of potentially fatal diseases such as hypoglycemic encephalopathy [13, 14] and Jamaican vomiting sickness [15–19] in humans and atypical myopathy in horses [20–24] and deers [25, 26]. Maple trees are especially abundant in central Europe and the USA, which may pose a risk of animal intoxication following ingestion of maple seeds and seedlings. Previous reports have indicated that these toxins could pass into the milk of mare [27, 28] and cows [29], consequently predisposing humans to a health risk if contaminated milk is ingested.

HGA and MCPrG are protoxins, and their toxicity is therefore attributed to their bioactivation through the metabolism into the coenzyme A (CoA) adducts of methylenecyclopropylacetic acid (MCPA) and methylenecyclopropylformic acid (MCPF), respectively. MCPA-CoA and MCPF-CoA block the enzymes necessary for the beta oxidation of fatty acids leading to the accumulation of fat esters that damage muscle cell membrane and thus triggering the symptoms of intoxication [24, 30, 31]. In addition, MCPA-CoA and MCPF-CoA block the enzymes involved in hepatic gluconeogenesis leading eventually to hypoglycemia after the hepatic glycogen stores are depleted [32, 33].

**Fig. 1:**
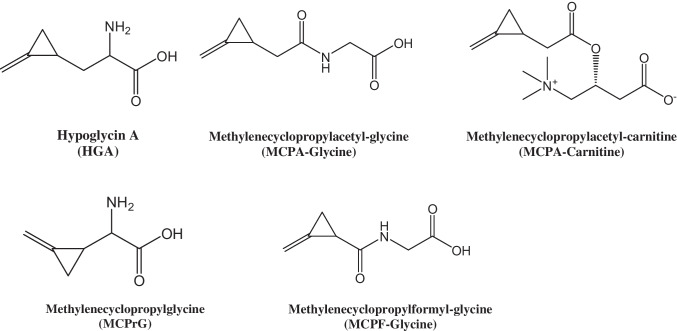
Chemical structures of the toxins and their metabolites investigated in this study

MCPA-CoA and MCPF-CoA are finally metabolized to their respective glycine and carnitine derivatives, namely methylenecyclopropylacetyl-glycine (MCPA-glycine) and methylenecyclopropylacetyl-carnitine (MCPA-carnitine) and methylenecyclopropylformyl-glycine (MCPF-glycine) and methylenecyclopropylformyl-carnitine (MCPF-carnitine). The structures of HGA, MCPrG, and their metabolites are shown in Fig. 1. The determination of HGA, MCPrG, and their glycine and carnitine metabolites in blood and urine is a useful tool for screening for the potential exposure to these toxins and probably also for prevention of further exposure in the respective animals [8, 22]. In addition, HGA, MCPA-glycine, MCPA-carnitine, MCPF-glycine, and MCPF-carnitine have been detected in some commercial horse milk samples [28]. HGA excretion in milk of other species has been also demonstrated by the detection of HGA in nursing lambs’ [34] and cow’s milk [29]. The evaluation of possible health risks to humans calls for reliable data on the presence and levels of HGA and MCPrG in milk.

Several LC–MS/MS methods for the quantification of HGA, MCPrG and their glycine and carnitine metabolites in urine and milk have been reported. The majority of these methods involved pre-column derivatization with butanol (3N HCl in *n*-butanol) [22, 28, 35] or fluorenylmethoxycarbonyl (Fmoc) [29]. Few methods have demonstrated the LC–MS quantification without derivatization [36]. To date, there is no method for the quantification of HGA, MCPrG, and their glycine and carnitine metabolites in milk validated according to, for example, the Directorate-General for Health and Food Safety (DG SANTE) guidelines [37] as required for food safety purposes in the EU.

In this work, simple, sensitive ultra-performance liquid chromatography tandem mass spectrometry (UPLC–MS/MS) methods without derivatization for the quantification of HGA, MCPrG, MCPA-glycine, MCPF-glycine, and MCPA-carnitine in cow’s milk and urine were developed and validated. In addition, the stability of HGA and MCPrG in stored milk has been assessed over 40 weeks. Finally, the validated method was applied for the screening of farm milk samples for the presence of HGA, MCPrG and their glycine and carnitine metabolites.

## Materials and methods

### Chemicals and standards

(S)-hypoglycin A (HGA, purity 85%), α-(methylenecyclopropyl)glycine (MCPrG, 97%), and MCPA-carnitine (97%) standards were purchased from Toronto Research Chemicals (Toronto, Canada). MCPA-glycine (97%) and MCPF-glycine (97%) standards were purchased from IsoSciences (Ambler, PA, USA). Although the purity of HGA standard (85%) is relatively low, the analysis showed that it does not contain any of the related analytes. Acetonitrile (ACN), methanol (MeOH), ethylenediaminetetraacetic acid (EDTA), formic acid (FA), and ammonium formate (NH_4_COOH) were of LC–MS grade and purchased from Merck (Darmstadt, Germany). C-_18_ material (Polygoprep 300-30C_18_) was purchased from Macherey–Nagel (Düren, Germany). Double-deionized water was obtained using a Milli-Q system from Merck (Merck Millipore, Darmstadt, Germany).

### Milk and urine sampling

Blank samples for method validation: Blank raw tank milk and urine samples were collected from cows at the BfR experimental farm in Berlin, Germany. The samples were stored at − 20 °C. Urine samples were collected during spontaneous micturition, carefully avoiding fecal contamination.

Farm milk samples: Overall, 35 commercial dairy farms providing pasture for their lactating cattle were sampled individually. The sampling plan covered different locations in Northern Germany (Schleswig–Holstein) as well as different production schemes (18 organic, 17 conventional). In order to compare seasonally different feeding regimes, 33 of these dairy farms were sampled twice. The first sample was obtained during the grazing period in summer and the second in winter during feeding of preserved feed. Thus, a total of 68 individual milk samples were taken from 35 different farms. The milk was sampled from the bulk tank after thorough mixing or, in the case of 7 ecological farms, from the self-service milk vending station. More information about the farms and sampling are found in Table S1. Milk was aliquoted to 30 mL portions into polypropylene screw cap tubes (Sarstedt, Nümbrecht, Germany) and stored at − 20 °C until analysis.

### Sample preparation

#### Milk

**Fig. 2:**
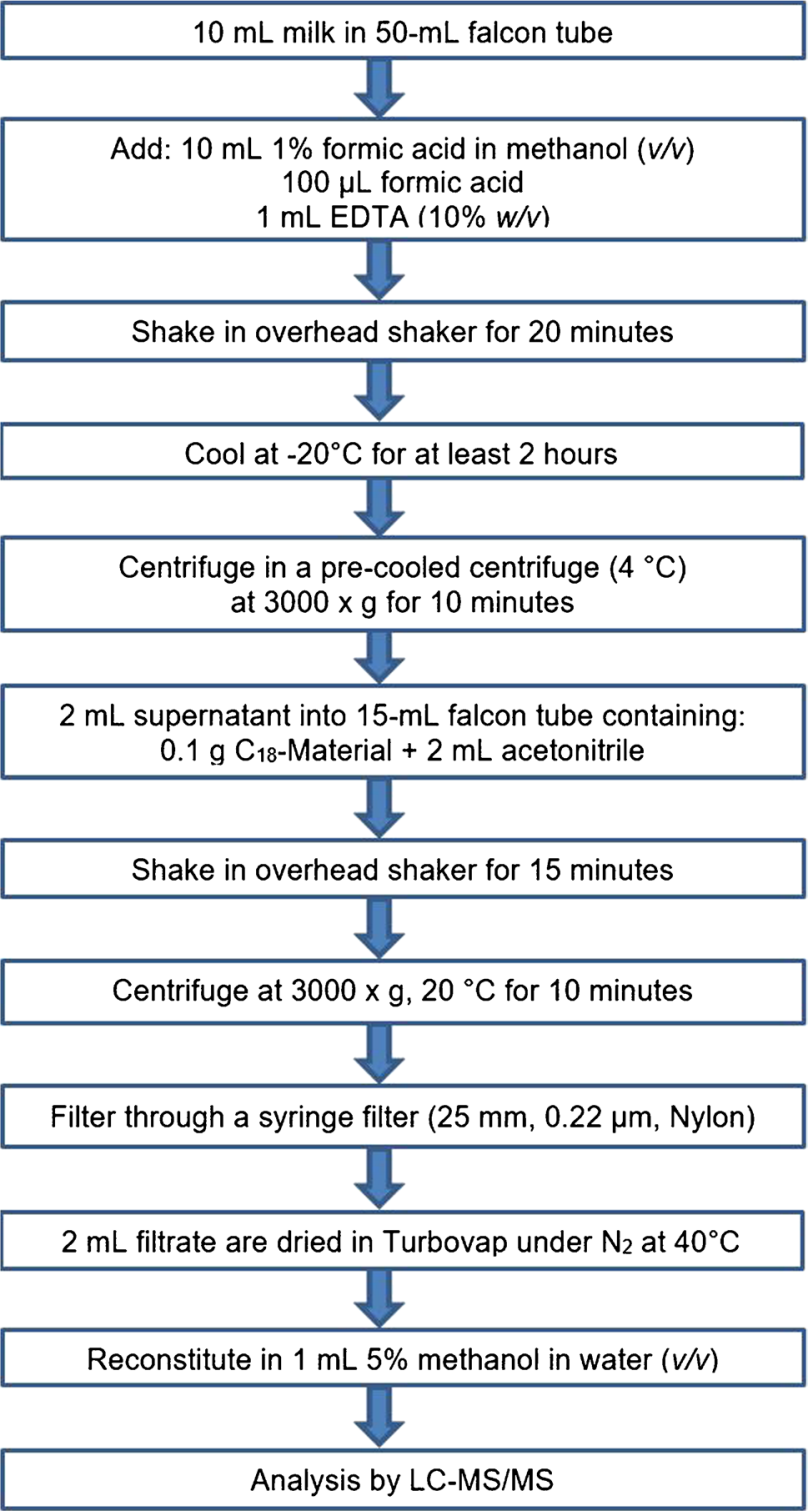
Extraction of HGA, MCPrG and their metabolites from raw milk

The sample preparation is summarized in Fig. 2. Apparatus: Overhead shaker: Reax 2, Heidolph Instruments (Schwabach, Germany); Centrifuge: Heraeus Megafuge 16, Thermo Fisher Scientific (Waltham, USA); Syringe Filter: Perfect-Flow, Wicom (Heppenheim, Germany); Turbovap: TurboVap LV, Zymark (MA, USA).

#### Urine

A dilute-and-shoot approach has been used. Urine was vortexed and centrifuged for 5 min at 13,500 × g (Centrifuge 5424 R, Eppendorf, Hamburg, Germany). The supernatant was diluted with 5% MeOH in water to a creatinine concentration of 0.1 mg/dL and then analyzed by LC–MS/MS. The determination of creatinine concentration was performed at an accredited medical analytics laboratory (Labor 28 GmbH, Berlin, Germany).

### Stock and working standard preparation

Stock HGA, MCPrG, MCPA-glycine, MCPF-glycine, and MCPA-carnitine standard solutions (0.1 mg/mL) were prepared in 50% ACN in water (v/v). A working standard mixture (1.0 µg/mL) was prepared by mixing stock solutions and dilution with 5% MeOH in water (v/v). For calibration, a series of solutions at 0.5, 1, 2, 3, 4, 5, 10, 25, 50, and 100 ng/mL each were prepared in 5% MeOH and blank extract (matrix-matched calibration).

### LC–MS/MS instrumentation and measurements

The LC–MS/MS analysis of extracted samples was performed in multiple reaction monitoring (MRM) mode as described elsewhere [11] using an Agilent 1290 Infinity II UPLC system (Agilent Technologies, Waldbronn, Germany) coupled to a Q-Trap 6500 + mass spectrometer (AB Sciex Germany GmbH, Darmstadt, Germany) equipped with an IonDrive™ Turbo V electrospray ionization (ESI) source. Chromatographic reversed-phase (RP) separation with 10 μL injection volume was achieved on a Waters Acquity UPLC BEH C18 column (150 × 2.1 mm, 1.7 μm particle size) at a flow rate of 0.3 mL/min and a column oven temperature of 40 °C. The binary mobile phase consisted of 5 mM ammonium formate and 0.1% FA in water (eluent A) and methanol (eluent B). MS detection was conducted using positive ionization mode. The MRM transitions and MS/MS conditions are shown in Table 1. The proposed structures of the product ions are shown in Figs. S1–S5.

**Table 1:** Mass transitions and conditions for LC–MS/MS quantification of HGA, MCPrG, MCPA-glycine, MCPF-glycine, and MCPA-carnitine in cow’s milk and urine

Analyte	Precursor ion (m/z)	DP (V)	EP (V)	Product ions (m/z)	CE (V)	CXP (V)	Dwell Time (ms)	Expected RT (min)
MCPrG	128.0	35.8	4.45	64.8 (quant.) 92.0	25.8 20.5	7.0 14.0	80	1.79
HGA	142.0	15.0	2.60	73.9 (quant.) 46.2	11.0 18.5	8.0 9.0		3.13
MCPF-glycine	156.1	35.0	7.23	80.9 (quant.) 53.0	15.0 29.3	9.5 6.4		3.47
MCPA-glycine	170.1	33.0	11.50	73.8 (quant.) 68.9	19.4 15.6	8.2 7.5		3.83
MCPA-carnitine	256.2	32.0	13.50	84.9 (quant.) 197.1	27.0 20.0	13.5 18.5		3.66

*DP* declustering potential, *EP* entrance potential, *CE* collision energy, *CXP* collision cell exit potential, *RT* retention time.

### Method validation

Raw milk and urine samples were used as blank matrices for method validation. The absence of HGA, MCPrG, MCPA-glycine, MCPF-glycine, and MCPA-carnitine was confirmed. The method was validated according to the European Union SANTE/2021/11312 guidelines [37]. The method validation parameters and performance criteria are as follows:

- *Identification:* the retention time of the analyte in the extract should match that of the matrix-matched calibration standard with a tolerance of ± 0.1 min. Peaks of both MRM transitions in the extracted ion chromatograms must fully overlap. Ion ratio of MRM transitions from sample extracts should be within ± 30% of average of calibration standards from same sequence.
- *Linearity and range:* a series of matrix-matched standard (MMS) solutions in the range of 0.5–100 µg/L HGA, MCPrG, MCPA-glycine, MCPF-glycine, and MCPA-carnitine were evaluated. Deviation of back-calculated concentration from true concentration should be ≤  ± 20%.
- *Limit of detection (LOD), limit of quantification (LOQ):* LOD and LOQ were determined according to the EURL Guidance Document on the Estimation of LOD and LOQ for Measurements in the Field of Contaminants in Feed and Food [38] using spiked blank samples. In short, 10 independent spiked blank samples are analyzed. The standard deviation of signal values of these 10 spiked blanks is used for the estimation of LOD and LOQ as follows:
  $$LOD=3.9*\frac{{\varvec{S}}y,b}{{\varvec{b}}}$$

***S***_*y,b:*_ Standard deviation of the blank signals.

***b:*** Slope of the calibration curve

$$LOQ=3.3*LOD$$

- *Recovery:* four quality control (QC) samples were prepared by spiking blank samples. The QC levels for milk were: lowest validated level (LVL, 2.5 µg/L), low (QCL, 5 µg/L), medium (QCM, 50 µg/L), and high (QCH, 150 µg/L). For urine, the QC levels were LVL (100 µg/L), QCL (500 µg/L), QCM (2500 µg/L), and QCH (7500 µg/L). The average recovery for each QC level should be within 70–120%.
- *Precision:* repeatability (intra-day precision, RSD_r_) and within-laboratory reproducibility (inter-day precision, RSD_wR_) were determined for the QC samples. RSD_r_ and RSD_wR_ for each QC level should be ≤ 20%.
- *Matrix effect:* the response of the MMS solutions was compared to that of standard solutions prepared in methanol.
- *Stability:* long-term stability of HGA and MCPrG in raw milk was tested. Two QC samples (5 and 50 µg/L) were prepared by spiking blank raw milk and stored at − 20 °C. Aliquots of the QC samples were prepared and analyzed immediately after spiking and after 3, 5, 8, 24, and 40 weeks of storage. The stability was calculated as the % recovery of the calculated concentrations of the stored QC samples as compared to those obtained with freshly prepared ones (reference value at day 0).

### Data analysis

LC–MS/MS data evaluation was performed with MultiQuant Software, ver. 3.0.2 (AB Sciex Germany GmbH, Darmstadt, Germany).

## Results and discussion

### Method performance and validation

**Fig. 3:**
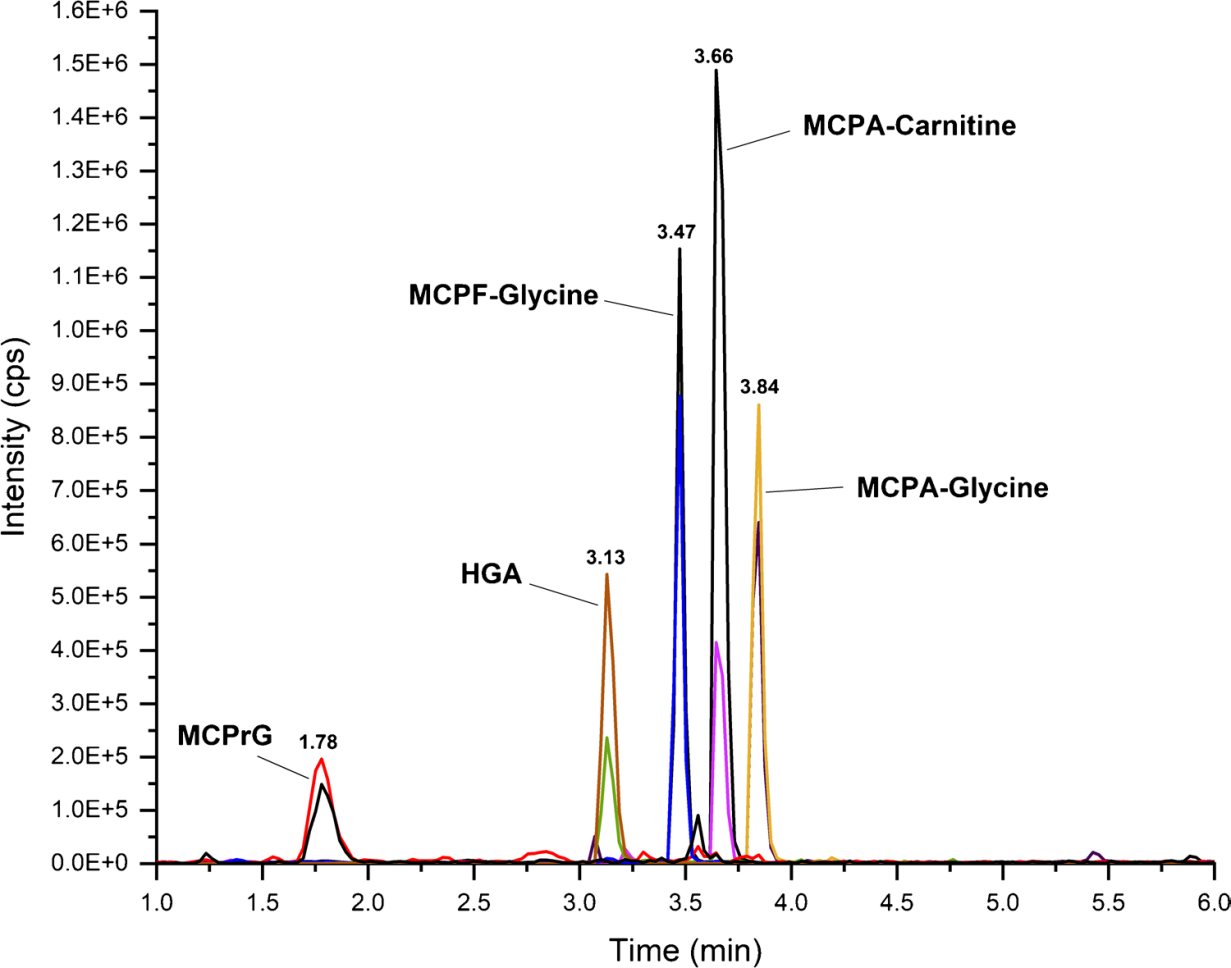
Overlaid MRM extracted ion chromatograms of HGA, MCPrG, MCPA-glycine, MCPF-glycine, and MCPA-carnitine in spiked cow’s raw milk

**Fig. 4:**
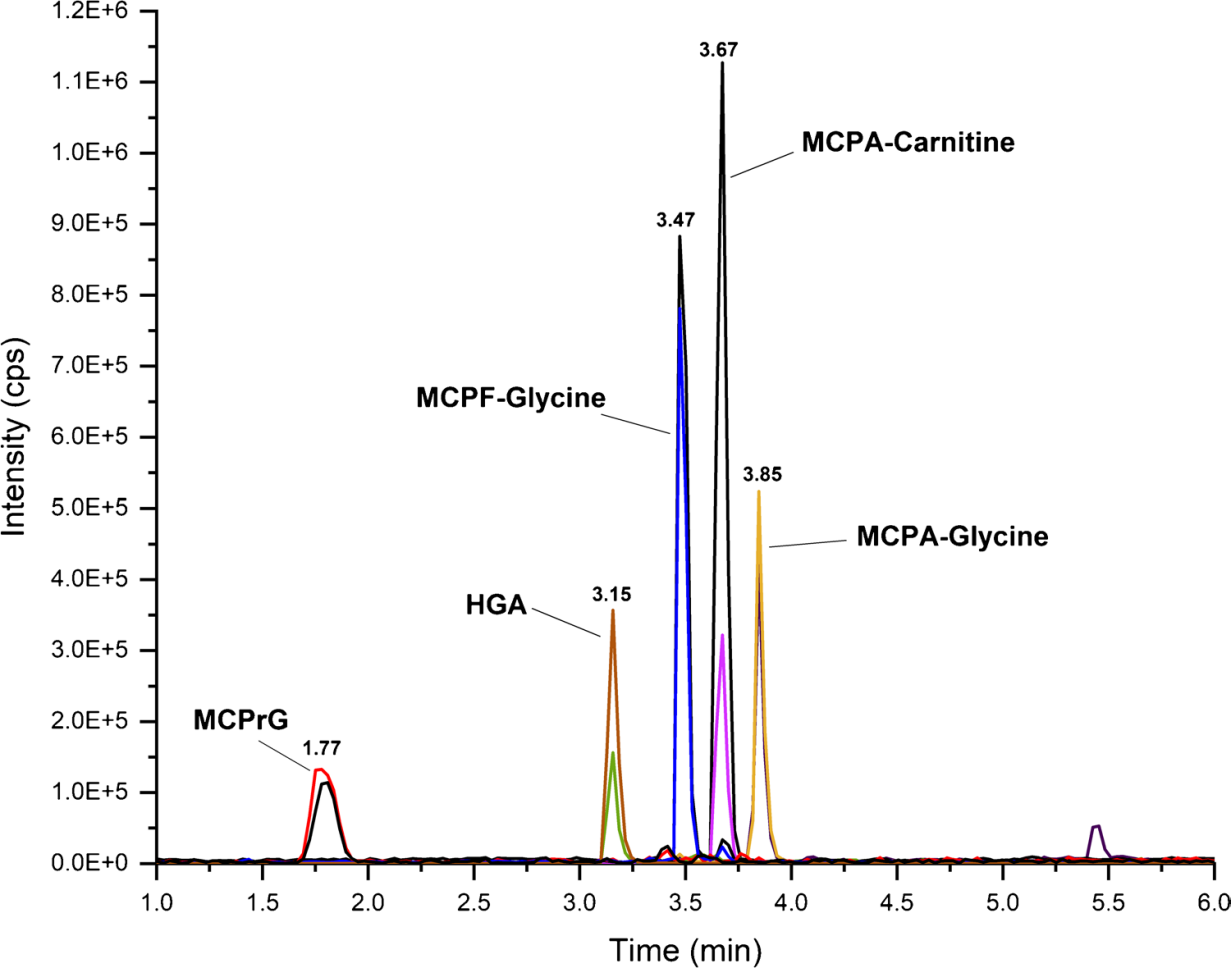
Overlaid MRM extracted ion chromatograms of HGA, MCPrG, MCPA-glycine, MCPF-glycine, and MCPA-carnitine in spiked cow’s urine

In this work, sensitive LC–MS/MS methods without derivatization were developed and validated for the quantification of HGA, MCPrG, MCPA-glycine, MCPF-glycine, and MCPA-carnitine in cow’s raw milk and urine samples. The MRM extracted ion chromatograms of these analytes in milk and urine are shown in Fig. 3 and Fig. 4, respectively. The chromatographic run-time was 10 min. The validation results were generally meeting validation requirements according to the SANTE guidelines and are summarized in Table 2 and Table 3 for milk and urine, respectively.

**Table 2:** Method validation parameters for the determination of HGA, MCPrG, MCPA-glycine, MCPF-glycine, and MCPA-carnitine in cow’s raw milk. LOD and LOQ are determined using spiked blank material

Parameter		HGA	MCPrG	MCPA-glycine	MCPF-glycine	MCPA-carnitine
Calibration range *(µg/L)		0.5–100 (1.06–211)	2–100 (4.2–211)	0.5–100 (1.06–211)	0.5–100 (1.06–211)	0.5–10 (1.06–21.1)
Correlation coefficient (***r***)		0.9973	0.9978	0.9989	0.9982	0.9998
LOD (µg/L)		0.34	2.63	0.30	0.33	0.23
LOQ (µg/L)		1.12	8.67	0.99	1.09	0.75
Recovery (%)	2.5 µg/L	89	< LOQ	100	91	103
	5 µg/L	98	101	106	99	n/d
	50 µg/L	98	98	102	96	n/d
	150 µg/L	96	97	97	94	n/d
Repeatability (RSD_r_) (%)	2.5 µg/L	11.5	< LOQ	6.2	6.3	0.6
	5 µg/L	9.1	9.0	7.1	4.3	n/d
	50 µg/L	4.8	8.0	5.2	5.1	n/d
	150 µg/L	5.9	6.3	6.8	2.4	n/d
Within**-**laboratory reproducibility (RSD_wR)_ (%)	2.5 µg/L	18.6	< LOQ	7.4	15.6	12.4
	5 µg/L	11.4	8.8	7.5	6.4	n/d
	50 µg/L	6.9	10.9	6.7	7.2	n/d
	150 µg/L	7.0	6.5	6.0	7.5	n/d
Matrix effect (%)		69	55	54	43	59

*Concentration in the matrix-matched calibration standards. Values in parentheses are the equivalent concentrations in milk samples (µg analyte/L milk, dilution factor of 2.11).

*n/d* not determined.

**Table 3:** Method validation parameters for the determination of HGA, MCPrG, MCPA-glycine, MCPF-glycine, and MCPA-carnitine in cow’s urine. LOD and LOQ are determined using spiked blank material

Parameter		HGA	MCPrG	MCPA-glycine	MCPF-glycine	MCPA-carnitine
Calibration range *(µg/L)		0.5–100 (50–10,000)	0.5–100 (50–10,000)	0.5–100 (50–10,000)	0.5–100 (50–10,000)	0.5–10 (50–1000)
Correlation coefficient (***r***)		0.9996	0.9999	0.9997	0.9995	0.9990
LOD (µg/L)		20.7	33.2	23.9	12.6	15.8
LOQ (µg/L)		68.2	109.5	78.9	41.6	52.3
Recovery (%)	100 µg/L	85	101	97	87	104
	500 µg/L	100	92	97	98	99
	2500 µg/L	99	95	98	102	n/d (> linear range)
	7500 µg/L	97	98	99	98	n/d (> linear range)
Repeatability (RSD_r_) (%)	100 µg/L	6.9	9.0	6.7	4.1	4.2
	500 µg/L	0.90	4.9	8.9	2.2	2.3
	2500 µg/L	2.6	1.3	3.1	2.5	n/d (> linear range)
	7500 µg/L	3.3	0.50	1.0	0.9	n/d (> linear range)
Within**-**laboratory reproducibility (RSD_wR)_ (%)	100 µg/L	10.6	12.6	10.0	11.9	5.7
	500 µg/L	3.9	10.3	8.5	6.1	6.5
	2500 µg/L	5.2	4.5	4.0	4.5	n/d (> linear range)
	7500 µg/L	2.6	2.3	1.9	2.4	n/d (> linear range)
Matrix effect (%)		108	94	53	103	103

*Concentration in the matrix-matched calibration standards. Values in parentheses are the equivalent concentrations in urine samples (µg analyte/L urine, dilution factor of 100).

*n/d* not determined.

### Linearity

In milk, the method is linear in the range of 0.5–100 µg/L (equivalent to 1.06–211 µg analyte/L milk, dilution factor of 2.11) for HGA, MCPA-glycine and MCPF-glycine. The linearity of MCPrG and MCPA-carnitine in milk are, however, in the range of 2–100 µg/L (4.2–211 µg MCPrG/L milk) and 0.5–10 µg/L (1.06–21 µg MCPA-carnitine/L milk). In urine, the method is linear in the range of 0.5–100 µg/L (equivalent to 50–10,000 µg analyte/L urine, dilution factor of 100) for HGA, MCPrG, MCPA-glycine, and MCPF-glycine. The linearity of MCPA-carnitine in urine is in the range of 0.5–10 µg/L (50–1000 µg MCPA-carnitine/L urine). It has to be emphasized that the validation results in urine (QC samples prepared by dilute-and-shoot to a creatinine concentration of 0.1 mg/dL) are calculated based on a blank urine sample with an initial creatinine concentration of 10 mg/dL (therefore a dilution factor of 100). Additional calculations are required for samples with other creatinine values. All matrix-matched calibration levels have fulfilled the criterion of within ± 20% deviation from the true concentrations using back-calculation.

### Sensitivity

The LOD and LOQ for HGA, MCPrG, MCPA-glycine, MCPF-glycine, and MCPA-carnitine in milk and urine are summarized in Table 2 and Table 3, respectively. The LOQ presented here for HGA in milk (1.12 µg/L) is noticeably lower than the limit of detection reported by Bochnia et al. (9 µg/L) [29]. In urine, the LOQ for MCPA-glycine and MCPF-glycine of the proposed method were 78.9 and 41.6 µg/L, respectively, which are lower than the lowest limit reported in human urine by Isenberg et al. (100 µg/L) [36].

### Accuracy and precision

Good values for the recovery (within 70–120%), repeatability (RSD_r_ ≤ 20%), and within laboratory reproducibility (RSD_wR_ ≤ 20%) were obtained for all analytes at the QC levels in milk and urine (Table 2 and Table 3). No correction of the values for the recovery has been done in the calculation of the other parameters because the recovery values were all close to 100% (89–106% and 85–104% in milk and urine, respectively) indicating no relevant loss during cleanup. The lowest validated level according to SANTE/2021/11312 guidelines is therefore: in milk, 2.5 µg/L for HGA, MCPA-glycine, MCPF-glycine, and MCPA-carnitine and 5 µg/L for MCPrG. While in urine, the lowest validated level is 100 µg/L for all analytes.

### Matrix effect

In milk, the matrix effects for the analytes are in the range of 43–69%, indicating signal suppression (Figs. S6–S10). The dilute-and-shoot of urine samples has reduced matrix effects for HGA, MCPrG, MCPF-glycine, and MCPA-carnitine (matrix effects in the range of 94–108%). However, a relatively stronger signal suppression in urine exists for MCPA-glycine (matrix effect was 53%) (Figs. S11–S15). Although the dilution approach showed good validation results in urine (Table 3), it, however, raises the LOQ of the method. Therefore, the dilution approach is only suitable for samples that contain sufficiently high levels of the analytes.

The use of matrix-matched calibration standards eliminates the need for the correction of matrix effects. This is demonstrated by the good recoveries at all QC levels (matrix effects are covered by the recovery criteria) (Table 2 and Table 3). Isotope-labeled internal standards as a means to correct for matrix effect were not available for the compounds investigated in the present study.

### Stability

**Fig. 5:**
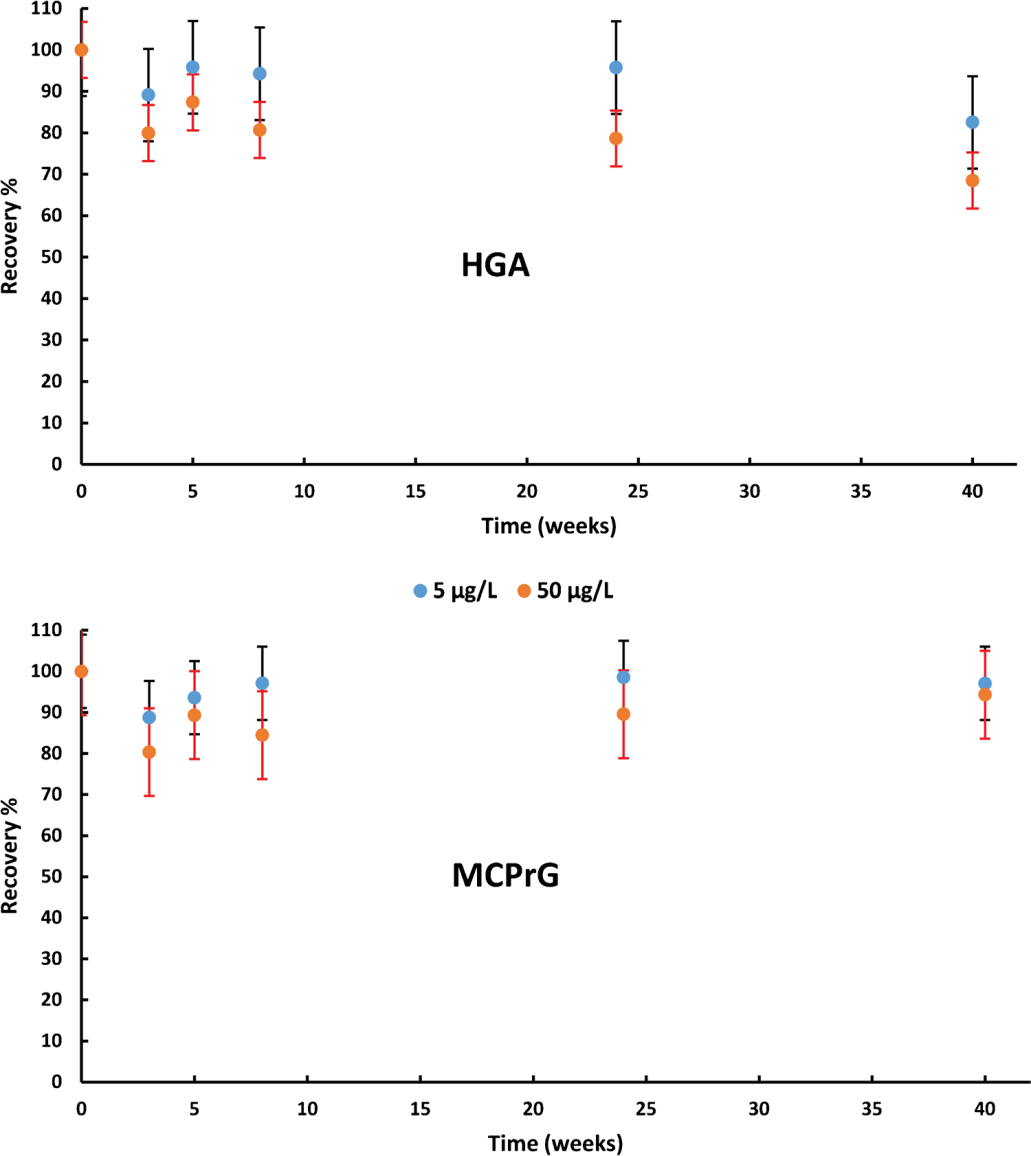
Long-term stability of HGA (upper panel) and MCPrG (lower panel) in spiked milk samples stored at − 20 °C. QC samples at 5 and 50 µg/L have been routinely analyzed. The data are presented as the % ratio ± standard deviation of the calculated concentrations of the stored QC samples as compared to those obtained with freshly prepared ones (reference value at day 0)

Over 40 weeks, the stability of HGA and MCPrG in milk stored at − 20 °C was demonstrated at 2 QC levels (5 and 50 µg/L) (Fig. 5). The decrease in concentration seems to be related to the initial freezing. Overall, the stability of HGA and MCPrG in milk was shown to be acceptable for the purpose of this study. Therefore, the proposed method could be used for the determination of HGA and MCPrG in preserved milk samples (providing proper storage at − 20 or − 80 °C).

### Application to farm milk samples

Reports on the presence of HGA in milk had raised some concern with view to a potential food safety risk. In order to get a better insight into the occurrence of HGA in milk and thus a potential exposure of consumers, the proposed validated method has been applied for the determination of HGA, MCPrG, and their metabolites in milk samples from 35 farms. The milk samples have been collected from individual farms, thus avoiding any dilution effects from pooling milk from contaminated source with non-contaminated milk during milk collection and processing at the dairy company. HGA, MCPF-glycine, MCPA-glycine, and MCPA-carnitine were not detected above LOD in any of the samples. Therefore, the samples can be considered free of any quantifiable amounts of HGA, MCPrG, and their metabolites. The results indicate that the presence of HGA in milk is not a widespread issue. However, there may be a seasonal and regional variability as the presence of maple seed, seedlings, or leaves in cow’s feed is the prerequisite for a potential HGA uptake and ultimately the transfer into milk. The presence of HGA and MCPrG in feed on the sampled farms cannot be excluded since the feed was not analyzed. Gonzales-Medina et al. proved the stability of HGA in sycamore seedlings stored in hay and silage hence cattle could get in contact with contaminated feed all over the year [39]. The lack of evidence of HGA, MCPrG and their metabolites in the farm milk samples could therefore also be due to microbial degradation processes of HGA and MCPrG in the rumen. Smith described ruminal detoxification of several plant compounds by gastrointestinal microbes [40].

### Conclusions

In conclusion, the methods proposed in this work allow the sensitive and reliable quantification of HGA, MCPrG, MCPA-glycine, MCPF-glycine, and MCPA-carnitine in cow’s raw milk and urine. The methods could be used for routine monitoring of these analytes. To the best of our knowledge, this is the first validated method for the quantification of HGA, MCPrG, and their metabolites in milk. Application to farm milk samples showed the absence of these analytes in all samples and thus that transfer of maple toxins into milk seems not to be a widespread issue.

## Supplementary Information

Below is the link to the electronic supplementary material.

Supplementary file1 (DOCX 627 KB)
