# Supplementary material for: A sensitive LC–MS/MS method for the quantification of the plant toxins hypoglycin A and methylenecyclopropylglycine and their metabolites in cow’s milk and urine and application to farm milk samples from Germany
Source: Anal Bioanal Chem. 2023 Mar 6;415(10):1933–42. doi: 10.1007/s00216-023-04607-9 (PMC10050036; doi:10.1007/s00216-023-04607-9)
Supplement: Supplementary file 1 — Supplementary file1 (DOCX 627 KB) [file 216_2023_4607_MOESM1_ESM.docx]

**A sensitive LC–MS/MS method for the quantification of the plant toxins hypoglycin A and methylenecyclopropylglycine and their metabolites in cow’s milk and urine and application to farm milk samples from Germany**

Ahmed H. El-Khatib^1^*, Julika Lamp^2^, and Stefan Weigel^1^

^1^ Department for Safety in the Food Chain, German Federal Institute for Risk Assessment (BfR), Max‑Dohrn‑Str. 8‑10, 10589 Berlin, Germany

^2^ Department Safety and Quality of Milk and Fish Products, Max Rubner-Institut, Federal Research Institute of Nutrition and Food, Hermann-Weigmann-Str. 1, 24103 Kiel, Germany

* Correspondence: ahmed.el-khatib@bfr.bund.de

**Table S1** Types of farms and sampling details for milk used in this study.

|  |  |  | **Herd performance (kg/day)** | | | **Herd size (lactating)** | | | **Grassland size (ha)** | | |
| --- | --- | --- | --- | --- | --- | --- | --- | --- | --- | --- | --- |
|  | Number | Sampling | Average | Min. | Max. | Average | Min. | Max. | Average | Min. | Max. |
| **Conventional farms** | 17 | Bulk tank | 9312 | 7500 | 10600 | 107 | 50 | 230 | 81 | 25 | 220 |
| **Ecological farms** | 11 | Bulk tank | 7104 | 3500 | 10000 | 92 | 25 | 450 | 77 | 18 | 200 |
|  | 7 | Self-service milk vending station | No information available | | | | | | | | |

**Ecological farms:** 7 farms were sampled at the self-service milk vending station without the manager. Therefore, the data on herd performance, herd and grassland size are only available for the 11 farms sampled at the bulk tank.

**Fig. S1** Fragmentation pathways of MCPrG.

**Fig. S2** Fragmentation pathways of HGA.

**Fig. S3** Fragmentation pathway of MCPF-Glycine.

**Fig. S4** Fragmentation pathways of MCPA-Glycine.

**Fig. S5** Fragmentation pathway of MCPA-Carnitine.

**Fig. S6** Determination of matrix effect. Calibration series of HGA standards in solvent and blank milk extract (matrix-matched standards, MMS).

**Fig. S7** Determination of matrix effect. Calibration series of MCPrG standards in solvent and blank milk extract (matrix-matched standards, MMS).

**Fig. S8** Determination of matrix effect. Calibration series of MCPA-glycine standards in solvent and blank milk extract (matrix-matched standards, MMS).

**Fig. S9** Determination of matrix effect. Calibration series of MCPF-glycine standards in solvent and blank milk extract (matrix-matched standards, MMS).

**Fig. S10** Determination of matrix effect. Calibration series of MCPA-carnitine standards in solvent and blank milk extract (matrix-matched standards, MMS).

**Fig. S11** Determination of matrix effect. Calibration series of HGA standards in solvent and diluted blank urine (matrix-matched standards, MMS).

**Fig. S12** Determination of matrix effect. Calibration series of MCPrG standards in solvent and diluted blank urine (matrix-matched standards, MMS).

**Fig. S13** Determination of matrix effect. Calibration series of MCPA-glycine standards in solvent and diluted blank urine (matrix-matched standards, MMS).

**Fig. S14** Determination of matrix effect. Calibration series of MCPF-glycine standards in solvent and diluted blank urine (matrix-matched standards, MMS).

**Fig. S15** Determination of matrix effect. Calibration series of MCPA-carnitine standards in solvent and diluted blank urine (matrix-matched standards, MMS).
